# Biological augmentation of anterior cruciate ligament reconstruction with bone marrow aspirate concentrate: a systematic review and meta-analysis of randomised controlled trials

**DOI:** 10.1007/s00264-024-06380-5

**Published:** 2024-11-22

**Authors:** Jae Yong Park, James Andrew Ng Hing Cheung, Dominik Todorov, Shin Young Park, Hayeon Lim, Eunjae Shin, Angelina Yoon, Joon Ha

**Affiliations:** 1 Faculty of Medicine, Imperial College London, Ayrton Rd, South Kensington, London, SW7 5NH UK; 2 Faculty of Medicine, Manchester University, Manchester, UK; 3 Faculty of Medicine, Kyungpook National University, Daegu, South Korea; 4 Faculty of Medicine, Monash University, Melbourne, Australia; 5 Foothills Medical Centre, University of Calgary, Calgary, Canada

**Keywords:** Anterior cruciate ligament reconstruction, Bone marrow aspirate concentrate, Biological augmentation, Patient-reported outcomes, Systematic review and meta-analysis

## Abstract

**Purpose:**

Biological augmentation of anterior cruciate ligament (ACL) reconstruction with bone marrow aspirate concentrate (BMAC) is gaining attention for its theoretical potential to enhance postoperative healing and recovery. However, its clinical benefits remain uncertain, and its high cost raises questions about efficacy. Hence, we systematically reviewed randomised controlled trials (RCTs) to evaluate the effectiveness of BMAC in ACL reconstruction.

**Methods:**

Our search included Cochrane, EMBASE, OVID, PubMed, and Scopus databases for RCTs evaluating the use of BMAC in ACL reconstruction. Primary outcomes focused on International Knee Documentation Committee (IKDC) scores and Lysholm scores. Secondary outcomes included MRI-related outcomes and postoperative complications. Statistical analysis was conducted using Review Manager 5.4 (Cochrane Collaboration), with heterogeneity assessed using Cochrane’s Q test and I^2^ statistics.

**Results:**

221 patients from five RCTs were included, with 109 (49.3%) receiving BMAC augmentation. Follow-up ranged from 11.05 to 24 months. No significant differences were found in postoperative IKDC scores between the BMAC and control groups at, three, six and 12 months. The BMAC group had significantly higher IKDC scores at 24 months; however, this difference was unlikely to be clinically significant. No significant differences were observed in postoperative Lysholm scores at 12 or 24 months. MRI-related outcomes suggested potential graft recovery improvement with BMAC, and complication rates were comparable between groups.

**Conclusion:**

In summary, biological augmentation with BMAC in ACL reconstruction does not significantly improve early patient-reported outcomes but offers potential benefits in graft recovery without increasing complication rates.

## Introduction

The anterior cruciate ligament (ACL) is a crucial intracapsular ligament within the knee joint that stabilises the knee, controls forward movement, and prevents excessive rotation of the tibia. ACL tears account for 50% of all knee injuries and affect more than 200,000 individuals in the United States annually [1]. This figure is on the rise, particularly in females and adolescents, due to their increased participation in sports [2–3]. The most common mechanism of an ACL tear is non-contact, often occurring during sudden landings, decelerations, or repeated pivoting movements [4].

Widely regarded as the gold standard for treating ACL tears, ACL reconstruction involves arthroscopically removing the damaged ligament and replacing it with a graft [5]. Various graft options are available, including autografts, where tissue is harvested from the patient, and allografts, which use donor tissue. While bone-patellar tendon-bone (BTB) and hamstring tendon (HT) grafts are commonly used, alternatives like the peroneus longus tendon have demonstrated non-inferiority with fewer donor-site complications [6].

Despite ACL reconstruction being the preferred treatment, it is limited by donor-harvest morbidity, a low rate of full recovery to sports, and incomplete microscopic healing of the graft [7]. To enhance postoperative healing and recovery, biological augmentation techniques, such as bone marrow aspirate concentrate (BMAC), can be used alongside ACL reconstruction [8].

BMAC is derived from human bone marrow, typically harvested from sites like the iliac crest, where bone marrow is abundant. After harvesting, the bone marrow aspirate is centrifuged to create a concentrated solution, which is then injected directly into the target tissue under sterile conditions [9]. BMAC contains a variety of multipotent cells, including mesenchymal stromal cells, haematopoietic stem cells, and vascular progenitors, which can help repair and regenerate damaged tissues [10–11]. Additionally, BMAC is rich in cytokines and growth factors with anti-inflammatory properties, crucial for cell maintenance and function [10–11].

Despite these theoretical advantages, there remains considerable debate about the clinical benefits of using BMAC for biological augmentation in ACL reconstruction. Given the substantial cost of BMAC, averaging around 3,000 USD [12], our systematic review and meta-analysis (SRMA) aims to provide a clearer understanding of its therapeutic benefits for patients considering this augmentation in ACL reconstruction. Furthermore, there are currently no SRMAs specifically focused on this intervention. Therefore, our SRMA not only aims to be the first of its kind but also intends to synthesise and present the highest level of evidence by exclusively utilising level-one randomised controlled trial (RCT) studies.

## Methods

The SRMA was conducted following the Cochrane Collaboration Handbook for Systematic Review of Interventions and the Preferred Reporting Items for Systematic Reviews and Meta-Analysis (PRISMA) Statement [13].

### Search strategy

The Cochrane, EMBASE, OVID, PubMed, and Scopus databases were systematically searched across all fields from inception to August 9, 2024, using the search string: (ACL OR “anterior cruciate ligament” OR “ACL reconstruction”) AND (BMAC OR “bone marrow aspirate” OR “bone marrow concentrate”).

### Eligibility criteria and study selection

The inclusion criteria were as follows: (1) RCTs; (2) skeletally mature patients or patients aged 18 or older; (3) patients with partial or complete ACL tears undergoing primary ACL reconstruction; (4) use of any autograft or allograft; (5) incorporation of BMAC for biological augmentation; (6) minimum follow-up of three months; and (7) reporting of at least one primary or secondary outcome. Exclusion criteria were as follows: (1) use of synthetic grafts or xenografts; and (2) non-English publications. References from included studies and previous systematic reviews were manually reviewed for additional relevant studies. Additionally, a manual search was conducted to account for potentially relevant papers that were not indexed in the databases used. Four independent reviewers (J.Y.P., J.A.N.H.C., D.T., and S.Y.P.) conducted the literature search, performed quality assessments, and collaboratively reviewed the selected articles to extract relevant data. The SRMA protocol was registered on PROSPERO with the identifier #CRD42024565461 on July 15, 2024.

### Data extraction

The authors collected the following data from the studies: (1) baseline characteristics; (2) primary outcomes, including International Knee Documentation Committee (IKDC) scores and Lysholm scores measured before surgery and at three, six, 12, and 24 months postoperatively; and (3) secondary outcomes, including all magnetic resonance imaging (MRI)-related findings and complications at three, six, 12, and 24 months post-surgery.

### Quality assessment

Risk of bias was independently assessed by three authors (J.Y.P., D.T., and S.Y.P.), utilising the Risk of Bias tool for Randomised Trials (RoB-2) following Cochrane’s guidelines [14]. Any discrepancies were resolved through discussion with the senior author (J.H.).

### Data analysis

Continuous outcomes were analysed using mean differences (MD), and binary outcomes with risk ratios (RR), both with 95% confidence intervals (CI). The meta-analysis employed the DerSimonian and Laird random effects model. For studies lacking variability data, standard deviations (SD) were imputed by averaging SDs from other studies in the same meta-analysis, as per Cochrane’s guidelines. For studies providing only graphical data, numerical data was first requested from the corresponding author. If no response was received, three authors (J.Y.P., J.A.N.H.C., and D.T.) independently extracted the data using Plot Digitizer (SourceForge, Fremont, CA, USA), averaging the values according to Cochrane’s guidelines. Any inconsistencies were resolved in consultation with the senior author (J.H.). Statistical analyses were conducted using Review Manager 5.4 (The Cochrane Collaboration, London, UK). For secondary outcomes where meta-analysis was not possible, a narrative synthesis was performed.

### Assessment of heterogeneity

Heterogeneity was assessed using Cochrane’s Q test and I² statistics, with significance defined by p-values under 0.10 and I² over 25%, according to Cochrane’s guidelines.

## Results

### Literature search

**Fig. 1:**
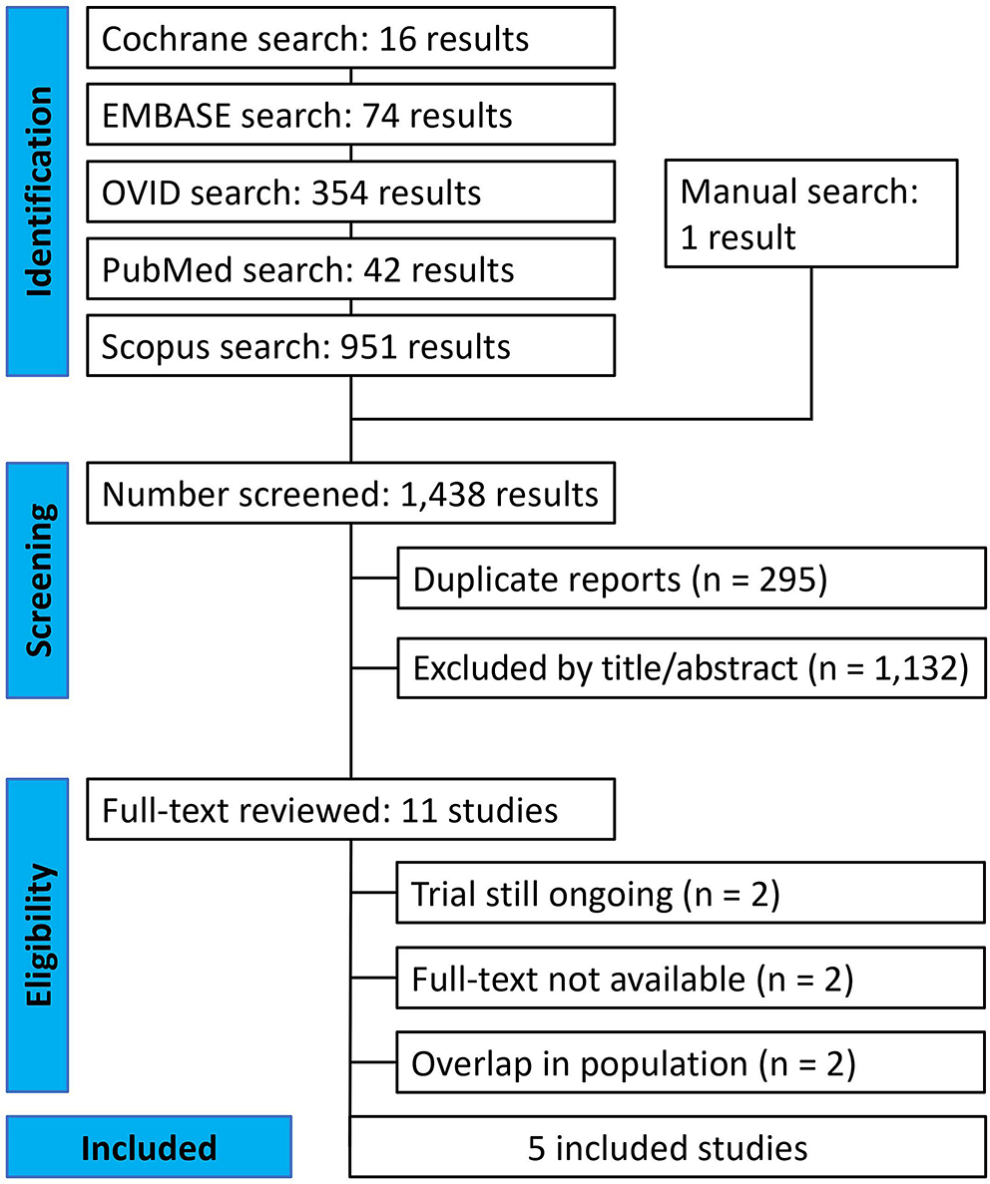
PRISMA flow diagram of study screening and selection

The literature search yielded 1,437 articles from various databases: Cochrane (*n* = 16), EMBASE (*n* = 74), OVID (*n* = 354), PubMed (*n* = 42), and Scopus (*n* = 951) (Fig. 1). One article was identified through handsearching. After removing 295 duplicates, three independent reviewers (J.Y.P., J.A.N.H.C., and S.Y.P.) screened the remaining articles, excluding 1,132 based on title or abstract. This left 11 papers for full-text review, of which six were excluded for not meeting the inclusion criteria. Any discrepancies were resolved through discussion with the senior author (J.H.). Ultimately, five RCTs were included in this SRMA [8, 15–18].

### Characteristics of the included studies and quality assessment

The baseline characteristics of the included studies (Table 1) involve a total of 221 patients undergoing ACL reconstruction, with or without BMAC augmentation. Among them, 109 (49.3%) patients received BMAC augmentation, while 112 (50.7%) underwent standard ACL reconstruction surgery without augmentation. Graft types, BMAC collection sites, and injection volumes varied across the studies. Additionally, three studies explored the combined effects of BMAC with other augmentation techniques. Follow-up periods ranged from 11.05 to 24 months.

**Table 1:** Baseline characteristics of the included RCT studies [8, 15–18]

Study	Journal	Date	Graft Type	Site of BMAC Collection	Volume of BMAC (mL) Injected	Other Augmentation Techniques	Patients, *n*(%)		Male, *n*(%)		Age (years)		Follow-Up (months)
							BMAC	Control	BMAC	Control	BMAC	Control	
Anz et al. [15]	The Orthopaedic Journal of Sports Medicine	2023	BTB autograft	DF, AIC, PIC	2	ACMW	10 (50.0)	10 (50.0)	4 (40.0)	5 (50.0)	29.8 ± 8.2	29.0 ± 6.8	12
			HT autograft				10 (50.0)	10 (50.0)	3 (30.0)	4 (40.0)	29.8 ± 8.2	25.3 ± 8.9	
Bhamare et al. [16]	Medical Journal of Dr. D.Y. Patil Vidyapeeth	2023	HT autograft	IC	3	None	15 (50.0)	15 (50.0)	NR	NR	NR	NR	24
Forsythe et al. [17]	Arthroscopy: The Journal of Arthroscopic and Related Surgery	2022	BTB allograft	AIC	2.5	None	36 (49.3)	37 (50.7)	15 (38.5)	18 (42.9)	36.3 ± 9.5	36.6 ± 8.8	24
Lavender et al. [8]	Arthroscopy: The Journal of Arthroscopic and Related Surgery	2024	QT autograft or allograft	PT	3	DBM, ST	29 (49.2)	30 (50.8)	18 (62.1)	15 (50.0)	22.8 ± 9.2	21.5 ± 7.7	24
Lin et al. [18]	Journal of Orthopaedic Surgery and Research	2024	HT autograft	PT	NR	PRP	9 (47.4)	10 (52.6)	5 (55.6)	6 (60.0)	27.1 ± 3.9	29.7 ± 9.9	11.05*

*The follow-up periods in the study by Lin et al. [18] were originally expressed in weeks, consequently these were approximately converted to months by dividing the number of weeks by 4.34525; ACMW: Amnion Collagen Matrix Wrap; BMAC: bone marrow aspirate concentrate; BTB: bone-patellar tendon-bone; DBM: demineralised bone matrix; DF: distal femur; HT: hamstring tendon; NR: not reported; PRP: platelet-rich plasma; PT: proximal tibia; QT: quadriceps tendon; ST: suture tape

Two of the five studies demonstrated a low risk of bias across all domains, resulting in an overall low risk (Table 2). In contrast, the other three studies had a high overall risk of bias due to a high risk in the “selection of the reported result” domain. Consequently, the predicted direction of bias likely favours the intervention.

**Table 2:** Risk of bias for the included RCT studies [8, 15–18]

Study	Bias from randomization process	Bias due to deviations from intended interventions	Bias due to missing outcome data	Bias in measurement of the outcomes	Bias in selection of the reported result	Overall risk of bias		
Anz et al. [15]	Low risk	Low risk	Low risk	Low risk	Low risk	Low risk		
Bhamare et al. [16]	Low risk	Low risk	Low risk	Low risk	High risk	High risk		
Forsythe et al. [17]	Low risk	Low risk	Low risk	Low risk	High risk	High risk		
Lavender et al. [8]	Low risk	Low risk	Some concerns	Low risk	High risk	High risk		
Lin et al. [18]	Low risk	Low risk	Low risk	Low risk	Low risk	Low risk		

Given the exclusive focus on RCTs in this SRMA and the absence of treatment crossover, an intention-to-treat analysis approach was employed to preserve the prognostic balance achieved through randomisation.

### Results from the study

**Fig. 2:**
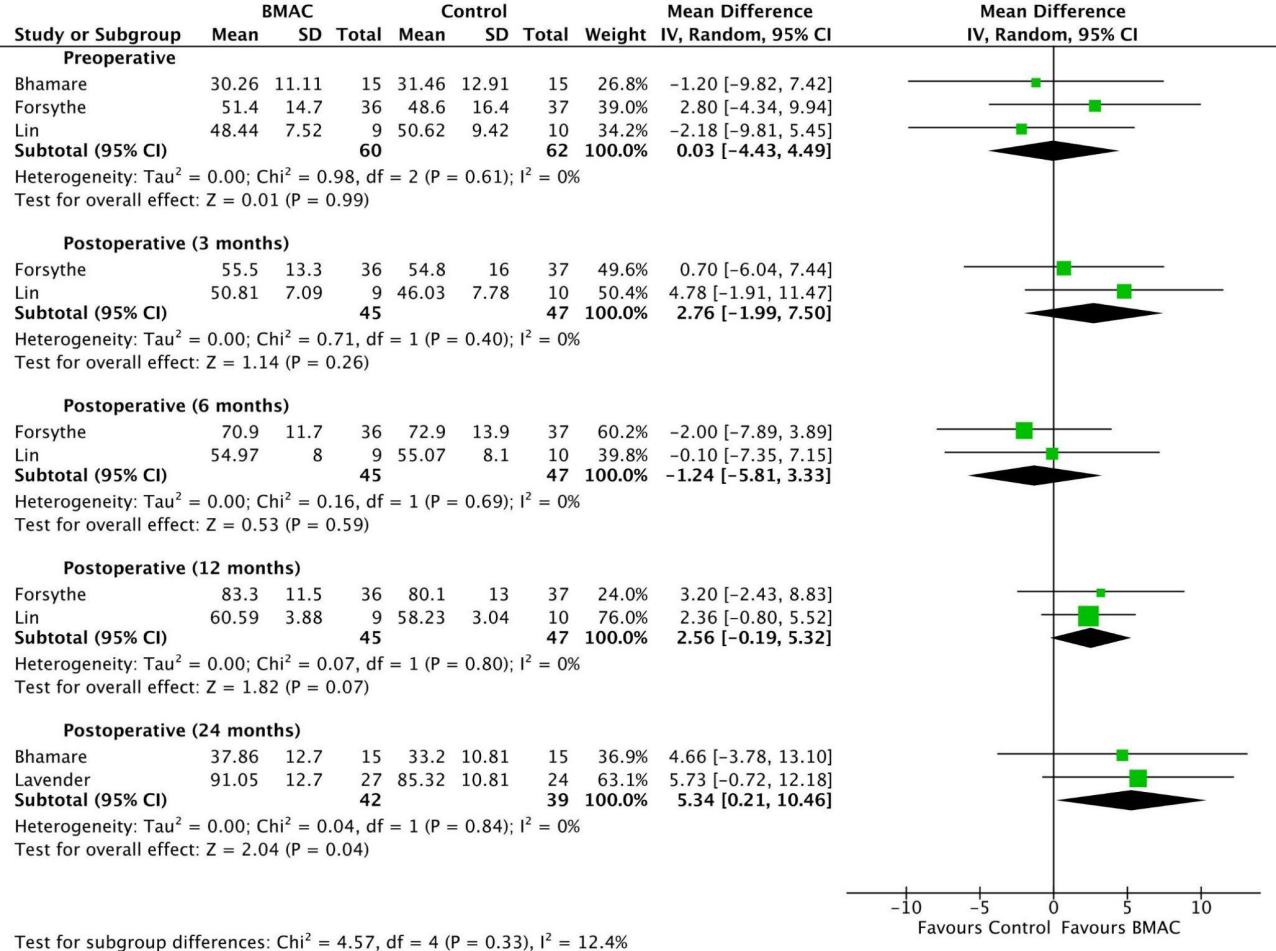
Comparison of IKDC scores over time: preoperative, 3, 6, 12, and 24 months postoperative

Patients who underwent biological augmentation of ACL reconstruction with BMAC had nearly identical preoperative IKDC scores (MD 0.03; 95% CI − 4.43 to 4.49; *p* = 0.99; I^2^ = 0%) (Fig. 2) compared to those who did not receive BMAC. This difference was not statistically significant, as indicated by a p-value greater than 0.05 and a 95% CI that includes 0, the value of no difference. At 3 months post-surgery, the BMAC group had higher IKDC scores (MD 2.76; 95% CI − 1.99 to 7.50; *p* = 0.26; I^2^ = 0%), although this difference was also not statistically significant. In contrast, at six months after surgery, the IKDC scores were lower in the BMAC group (MD − 1.24; 95% CI − 5.81 to 3.33; *p* = 0.59; I^2^ = 0%) compared to the control group, yet this difference was not statistically significant either. At 12 months post-surgery, the IKDC scores were higher in the BMAC group (MD 2.56; 95% CI − 0.19 to 5.32; *p* = 0.07; I^2^ = 0%), but this difference was not statistically significant. At 24 months post-surgery, the BMAC group also exhibited higher IKDC scores than the control group (MD 5.34; 95% CI 0.21 to 10.46; *p* = 0.04; I^2^ = 0%). However, this difference was found to be statistically significant.

**Fig. 3:**
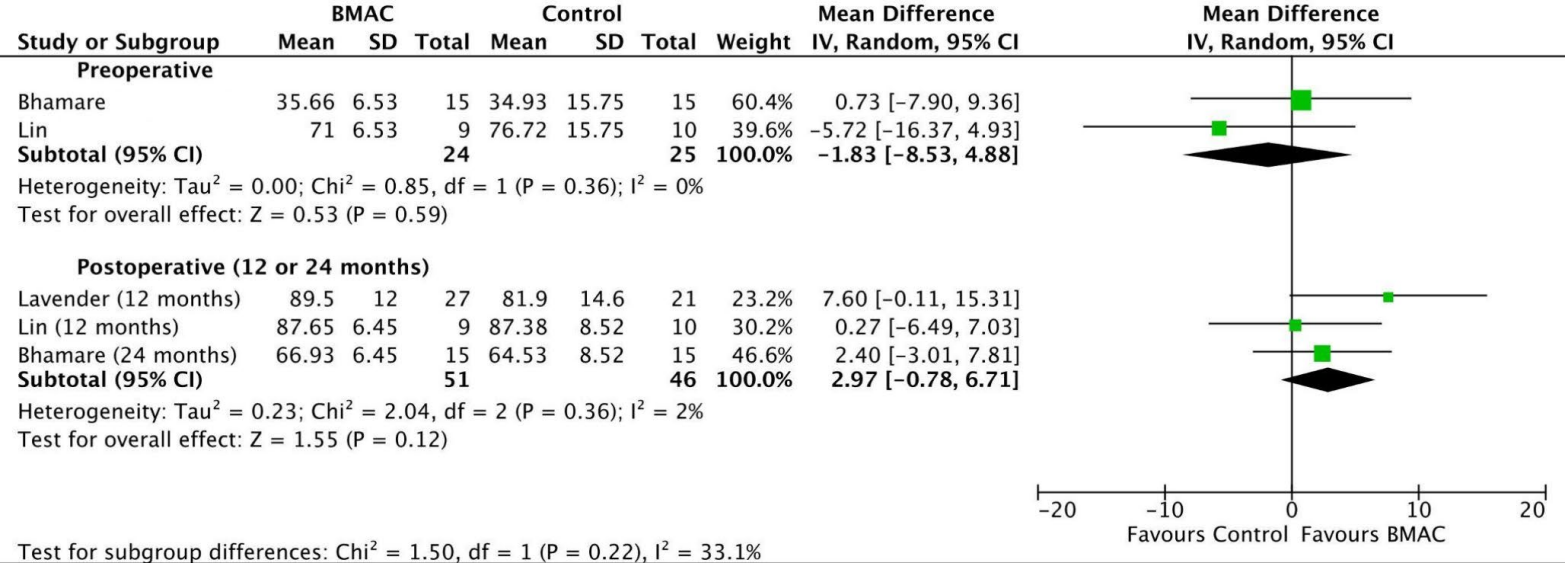
Comparison of Lysholm scores over time: preoperative and 12 or 24 months postoperative

The BMAC group demonstrated lower preoperative Lysholm scores (MD − 1.83; 95% CI − 8.53 to 4.88; *p* = 0.59; I^2^ = 0%) (Fig. 3) compared to the control group. On the other hand, the BMAC group showed higher Lysholm scores postoperatively at 12 or 24 months (MD 2.97; 95% CI − 0.78 to 6.71; *p* = 0.12; I^2^ = 2%). However, the differences in both preoperative and postoperative Lysholm scores were not statistically significant.

Four out of the five RCTs investigated MRI-related outcomes. However, due to the heterogeneity of these outcomes, a meta-analysis was not possible. Consequently, a narrative synthesis was conducted to assess the MRI-related findings of each individual study, investigating various postoperative changes in ACL grafts at distinct time points (Table 3):

1. Anz et al. utilised a linear mixed-effects model and found no significant differences between the BMAC and control groups in T2* relaxation values (M − 0.63; 95% CI − 1.89 to 0.63; *p* = 0.309) or graft volumes (M 135.1; 95% CI − 606 to 876.1; *p* = 0.706) within the BTB arm [15]. Similarly, in the HT arm, no significant differences were observed in T2* relaxation values (M − 0.89; 95% CI − 2.17 to 0.39; *p* = 0.162) or graft volumes (M − 395.81; 95% CI − 11,141.1 to 351.5; *p* = 0.280) between the groups.
2. Bhamare et al. conducted a single postoperative MRI assessment at nine months, assessing ligamentisation, tunnel widening, tunnel position, and complications, but did not provide any quantitative or qualitative data [16].
3. Forsythe et al. evaluated graft metabolic activity using signal intensity ratios (SIRs), defined as the ratio of ACL region signal intensity to patellar tendon signal intensity on sagittal T2-weighted MRI scans [17]. SIRs were measured across the superior, middle, and inferior thirds of the graft. The study found significantly higher SIRs in the inferior third of the graft in the BMAC group at three months compared to the control group (3.2 ± 2.2 vs. 2.1 ± 1.5; *p* = 0.023).
4. Lin et al. performed postoperative MRI scans at six, 12, 24, and 48 weeks, investigating graft maturation and tendon-bone interfacial healing [18]. Graft maturation was assessed by changes in graft signal intensity, and tendon-bone healing was evaluated by bone tunnel diameter. The BMAC group showed a delayed signal peak and higher graft signal at 24 weeks, though not statistically significant (*p* > 0.05). However, tendon-bone healing showed a significantly wider femoral bone tunnel diameter at 24 weeks in the BMAC group (*p* = 0.024). Additionally, peri-tunnel oedema was significantly higher in the BMAC group at 12 weeks compared to the controls (*p* < 0.05).

**Table 3:** Summary of reported MRI-related outcomes and findings for the included RCT studies [15–18]

Study	Group	Outcomes	Main Findings	Corresponding Values
Anz et al. [15]	BTB autograft	Mean T2* relaxation time value and volume for each graft	No significant differences in T2* relaxation values or graft volumes	T2* relaxation (M − 0.63; 95% CI − 1.89 to 0.63; *p* = 0.309), Graft volume (M 135.1; 95% CI − 606 to 876.1; *p* = 0.706)
	HT autograft			T2* relaxation (M − 0.89; 95% CI − 2.17 to 0.39; *p* = 0.162), Graft volume (M − 395.81; 95% CI − 11,141.1 to 351.5; *p* = 0.280)
Bhamare et al. [16]	HT autograft	Ligamentisation, tunnel widening, tunnel position, and complications	NR	NR
Forsythe et al. [17]	BTB allograft	Graft metabolic activity/SIR of superior, middle, and inferior third vs. patellar tendon	Higher SIRs in inferior third of graft at 3 months	SIRs (3.2 ± 2.2 vs. 2.1 ± 1.5; *p* = 0.023)
Lin et al. [18]	HT autograft	Graft maturation assessed by changes in graft signal intensity	Delayed signal peak and higher graft signal at 24 weeks, not statistically significant	Bone tunnel diameter (*p* = 0.024)
		Tendon-bone healing evaluated by bone tunnel diameter	Significantly wider femoral bone tunnel diameter at 24 weeks, higher peri-tunnel oedema at 12 weeks	Peri-tunnel oedema (*p* < 0.05)

BTB: bone-patellar tendon-bone; HT: hamstring tendon; SIR: signal intensity ratio; NR: not reported

A meta-analysis of complications was not possible due to the limited reporting. However, Lavender et al. reported stiffness in three BMAC patients and four controls at six weeks (Table 4) [8]. Additionally, two patients in each group underwent arthroscopic release and manipulation under anaesthesia (MUA) at six weeks, with one more BMAC patient undergoing MUA only. No ACL re-injuries were reported within 2 years post-surgery. In contrast, Anz et al. reported no complications, including infections, stiffness, persistent effusions, or reconstruction failures at the two year follow-up [15]. The remaining three studies did not mention postoperative complications [16–18].

**Table 4:** Summary of reported postoperative complications for the included RCT studies [8, 18]

Study	Time	Complication	BMAC Group, *n*(%)	Control Group, *n*(%)
Lavender et al. [8]	6 weeks	Stiffness	3 (10.3)	4 (13.3)
		Arthroscopic release and MUA	2 (6.9)	2 (6.7)
		MUA only	1 (3.4)	0 (0)
Lin et al. [18]	2 years	Any	0 (0)	0 (0)

BMAC: bone marrow aspirate concentrate; MUA: manipulation under anaesthesia

## Discussion

This SRMA included five RCTs with 221 patients, providing data on patient-reported outcomes using IKDC scores and Lysholm scores to assess the impact of BMAC augmentation in ACL reconstruction. The key findings are: (1) No significant differences between the BMAC and control groups in preoperative and postoperative IKDC scores at three, six and 12 months, but significantly higher postoperative IKDC scores in the BMAC group at 24 months; (2) No significant differences in preoperative and postoperative Lysholm scores at 12 or 24 months; (3) MRI results suggests potential for improved graft recovery in the BMAC group; (4) The reported complications suggests there may be no significant differences between the groups.

It is important to recognise that statistical significance does not always imply clinical significance. In our analysis, the only statistically significant result was a higher postoperative IKDC score in the BMAC group at 24 months (MD 5.34; 95% CI 0.21 to 10.46; *p* = 0.04; I^2^ = 0%). However, Franceschini et al. identified 17 minimal clinically important differences (MCIDs) for IKDC scores, using various calculation methods [19]. For ten of these MCIDs, the clinical significance of the postoperative IKDC score in the BMAC group at 24 months remains uncertain, as the CI includes those MCIDs. When applying the remaining MCIDs, the result would not be considered clinically significant, as the MCID exceeds the CI. Thus, our findings suggest there are no statistically or clinically meaningful differences between the BMAC and control groups.

Our SRMA had several limitations. Although all included studies focused on BMAC augmentation in ACL reconstruction, there was considerable variability across the five RCTs. These variations included differences in graft type, BMAC collection site, injection volume, and additional augmentation techniques. For example, Anz et al. used ACMW with BMAC [15], Lavender et al. combined BMAC with DBM and ST [8], and Lin et al. added PRP to BMAC [18]. This diversity makes it challenging to attribute the outcomes solely to BMAC or to its possible synergistic effects with the other techniques. Such variability may limit the generalisability of our findings regarding BMAC augmentation in ACL reconstruction. Therefore, more RCTs with standardised variables are needed to draw stronger conclusions and enable appropriate subgroup analyses.

Further limitations stemmed from incomplete or inconsistently reported data in some studies. For example, the Bhamare et al. study lacked variability data, requiring us to impute the SD by averaging the SDs from other studies in the meta-analysis [16]. While this approach may not fully capture the true variability, it was necessary to maintain the comprehensiveness of the analysis. Similarly, Lin et al. provided only graphical data [18], leading us to extract numerical data using Plot Digitizer (SourceForge, Fremont, CA, USA) after no response was received from the corresponding author. To minimise bias and improve precision, three authors (J.Y.P., J.A.N.H.C., and D.T.) independently extracted the data and averaged the values. Additionally, Lin et al. measured outcomes in weeks rather than months [18]. To ensure comparability, we aligned these outcomes with their closest monthly equivalents: 12 weeks as three months, 24 weeks as six months, and 48 weeks as 12 months. Although this conversion was necessary for consistency, it is important to acknowledge that the exact alignment between weeks and months may introduce slight discrepancies.

Our study was also limited by its relatively short follow-up period. While no statistically or clinically significant differences were observed in patient reported outcomes for BMAC augmentation of ACL reconstruction at three, six, 12, and 24 months, it is uncertain if these findings apply to longer follow-up periods. Therefore, RCTs with extended follow-up are needed to better understand long-term outcomes and potential complications.

Additionally, according to the RoB-2 tool for randomised trials, three of the five RCTs were rated as having a “high risk of bias”. Although this SRMA focuses on level-one RCTs, including studies with a “high risk of bias” may affect the reliability of our results. However, despite the predicted direction of bias favouring the intervention, the overall findings still indicate no statistically or clinically significant differences in the BMAC augmentation of ACL reconstruction. It is also important to note that the two RCTs with an overall “low risk of bias”—the studies by Anz et al. and Lin et al.—utilised BMAC augmentation in combination with ACMW and PRP, respectively [15, 18]. Thus, while their risk of bias is low, their outcomes may be influenced by the additional augmentation techniques employed, potentially limiting the generalisability of the findings to BMAC alone.

There was no significant heterogeneity observed in any of the postoperative outcomes (*p* < 0.10; I^2^ < 25%). However, the lack of heterogeneity may be due to low statistical power, likely related to the small sample size, which is a consequence of the limited number of studies on BMAC augmentation in ACL reconstruction. Consequently, additional RCTs on this intervention are necessary to draw more representative conclusions.

Further limitations arose from inconsistencies in postoperative MRI-related outcomes reported across the four studies. Although these outcomes are supported by both preclinical and clinical research for graft analysis [20–21], none of the studies employed a consistent set of measures, with most using only a single outcome. This variability, combined with the use of different MRI modalities such as T1 and T2 imaging, further complicates direct comparisons. As a result, despite aligning postoperative MRI assessments at comparable time points, the inconsistency in MRI-related outcomes prevented a quantitative analysis.

Given the lack of a standardised outcome measure for ACL graft evaluation, future research should prioritise the integration of standardised MRI-related outcomes. Recommended measures include T2* relaxation time for tissue composition, graft volume for structural evaluation, and SIRs for assessing metabolic activity [20–25]. Consistent use of these measures across studies would enhance the comparability and reliability of findings in postoperative ACL reconstruction imaging.

Moreover, the challenges of incomplete MRI data reporting in the Bhamare et al. study [16], and the reliance on manual annotation of MRI slides in the Anz et al. study [15], compromise the robustness and objectivity of their findings, limiting their usability. This highlights the need to potentially enhance MRI assessments as a surrogate for graft ligamentisation by incorporating additional methods like second-look needle arthroscopy. This approach could provide more objective and reliable results while potentially improving cost-effectiveness [26].

Despite our findings and their limitations, several studies suggest potential clinical benefits of BMAC in ACL reconstruction. For instance, a comparative study in rabbit models demonstrated enhanced regeneration using a combination of BMAC and growth factors [27]. Additionally, a systematic review by Hexter et al. indicates that multi-stem cell types, including BMAC, may improve biomechanical remodelling and strength in ACL reconstruction [28]. However, these studies are limited by small sample sizes and reliance on animal models, which do not fully replicate human biomechanical properties. Moreover, the clinical studies in the review primarily focused on growth factors like PRP, rather than BMAC. Furthermore, a non-controlled study by Centeno et al., involving 29 postoperative patients with symptomatic ACL tears, showed significant improvement in ACL integrity following treatment with platelet products and BMAC injections [29]. However, this study was limited by the lack of randomisation and control, potential placebo effects, missing data, and non-standardised rehabilitation protocols. Overall, while these studies do not specifically address ACL reconstruction, they offer valuable insights into the therapeutic potential of BMAC for ACL injuries.

## Conclusion

 The results of this SRMA suggest that biological augmentation with BMAC does not significantly improve ACL reconstruction outcomes, either statistically or clinically, as shown by similar early patient-reported outcomes. However, there is some evidence of potential improvements in graft recovery based on MRI-related outcomes, with minimal increase in complications. Despite these findings being based on level-one RCTs, it is important to recognise the limitations in the current literature, particularly the high risk of bias in some studies and the methodological variability across trials. Further studies with standardised protocols, larger sample sizes, and longer follow-up periods are needed to provide more definitive conclusions. Patients should carefully consider the potential benefits and costs of BMAC before pursuing this augmentation.

## Data Availability

Data and materials are available upon request by contacting the corresponding author.
